# Performance Effects of High Performance Work Systems on Committed, Long-Term Employees: A Multilevel Study

**DOI:** 10.3389/fpsyg.2022.825397

**Published:** 2022-03-14

**Authors:** Nikolaos Pahos, Eleanna Galanaki

**Affiliations:** ^1^Section of Economics, Technology and Innovation, Department of Values, Technology and Innovation, TU Delft, Delft, Netherlands; ^2^Department of Marketing and Communication, School of Business, Athens University of Economics and Business, Athens, Greece

**Keywords:** high performance work systems, employee performance, affective commitment, normative commitment, continuance commitment, employee tenure, social exchange theory, multilevel SEM

## Abstract

Even though effects of High Performance Work Systems (HPWS) on employee performance have been widely investigated, there is no consensus on how this link is achieved. Drawing on Social Exchange Theory (SET), this paper attempts to shed more light in this relationship by investigating the mediating role of affective, normative, and continuance commitment in the relationship between HPWS and employee performance. Moreover, the potential moderating role of employee tenure on the HPWS—organizational commitment link is examined. Using data from 342 subordinates and 115 supervisors from 111 service organizations in Greece, our multilevel analysis shows that affective commitment fully mediates the relationship between HPWS and employee performance. In addition, employee tenure positively moderates the relationships between HPWS and affective and normative commitment. The paper discusses theoretical implications and provides recommendations for practitioners.

## Introduction

Scholars in the field of Human Resource Management (HRM), have approached the field of HRM practices, by emphasizing on “High-commitment management practices” (Wood, 1996), “High-involvement work practices” (Guthrie, 2001), and “High performance work systems” (HPWS; Appelbaum et al., 2000). The effect of such practices on performance, both at the individual and at the organizational level, is an important field of research and provides significant findings (Evans and Davis, 2005; Aryee et al., 2012). However, despite the variety of empirical works in the field, little is known on how this relationship is achieved (Dorta-Afonso et al., 2021). For example, there is doubt about the exact mechanism through which the implementation of HRM practices leads to performance outcomes (Boxall et al., 2011). This issue is commonly referred to as the “black box” problem (Purcell et al., 2003) and refers to the human interactions and potential intervening factors within organizations, that might be crucial for enhancing HPWS’ performance outcomes.

Drawing on the different modes in theorizing HRM (Delery and Doty, 1996), in this study we adopt the universalistic approach and argue that HPWS may increase employee performance by acting synergistically and by contributing as systems (Fulmer and Ployhart, 2014). Unlike traditional micro-HRM research, strategic HRM (SHRM) research at the individual level “focuses on the influence of HR systems rather than a single HR practice on individual outcomes” (Jiang et al., 2013, p. 1453). Previous literature has attempted to understand the mechanisms through which HPWS are related to employee performance, and several dominant perspectives have been used to explain the “black box” at the business level of analysis in the strategic HRM literature (Messersmith et al., 2011; Jiang et al., 2013; Karadas and Karatepe, 2019). Recent empirical studies have also supported that HPWS affect individual performance, through the enhancement of specific employee attitudes and behaviors (Kehoe and Wright, 2013), such as knowledge (Michaelis et al., 2015), job satisfaction, turnover (Sun et al., 2007), and affective commitment (Chang and Chen, 2011; Takeuchi et al., 2018). This study attempts to shed more light in this conversation, by further exploring the mediating effect of organizational commitment in the HPWS—employee performance link. As a secondary objective, we explore the moderating effect of employee tenure on the link between HPWS and organizational commitment. Even though previous literature has explored links between tenure and employee outcomes (Ng and Feldman, 2010; Atatsi et al., 2021), it remains unknown how employee tenure can affect links between HPWS and different dimensions of organizational commitment. More specifically, we build on social exchange theory (SET; Blau, 1964) and argue that long-term employees will respond to the implementation of HPWS with more increased levels of organizational commitment, than short-term employees.

Our study contributes to the theory and the literature in many ways. First, while previous scholars have mainly focused on specific elements of organizational commitment (i.e., affective commitment), our study contributes to the existing literature by investigating the distinct mediating effects of affective, normative, and continuance commitment (Allen and Meyer, 1990), through a parallel mediation research model. Even though this relationship has been explored in the context of military service (Fragoso et al., 2019, 2021), little is known about how HPWS can affect different dimensions of organizational commitment in the workplace. Second, to our knowledge, this is the first study that investigates the moderating role of tenure on the link between HPWS and different aspects of organizational commitment. Third, we integrate the social exchange literature and HRM theoretical fields, by focusing on how employees reciprocate to a generally positive provision of HPWS, taking into consideration that the differing motives and preferences of short-term and long-term employees will result in different reciprocation patterns.

We examine the above relationships by utilizing data from 342 employees and 115 supervisors of 115 working groups, in 111 service organizations operating in Greece.

## Theoretical Framework and Hypotheses’ Building

### Affective, Normative, and Continuance Commitment

Organizational commitment is a widely investigated construct in the field of organizational psychology (Wang et al., 2020; Xu et al., 2020). Previous conceptualizations of organizational commitment may differ in terms of the psychological state of the commitment and the expected behaviors that emerge from it (Allen and Meyer, 1990). For the purpose of this study, we adopt the three-component model conceptualization of Meyer and Allen (1991), who distinguish between affective, normative, and continuance organizational commitment.

Affective commitment is considered to be an “affective or emotional attachment to the organization such that the strongly committed individual identifies with, is involved in, and enjoys membership in, the organization” (Allen and Meyer, 1990, p. 2). In other words, it is defined as the relative strength of an individual’s identification with a particular organization (Meyer et al., 1993) and it can be associated with personal, environmental, structural, and job-related characteristics, and work experiences (Mowday et al., 1979).

Normative commitment accounts for the employees’ loyalty to an organization, as a result of a mindset of obligation (Meyer and Parfyonova, 2010). People can develop normative commitment through socialization experiences that emphasize the importance of remaining loyal to one’s employer (Wiener, 1982), or because they feel the obligation to reciprocate potential benefits that they receive from the organization (Scholl, 1981).

Continuance commitment develops as employees recognize the costs (or lost side bets) that they would bear, if they were to leave the organization (Meyer et al., 1993). In addition, this type of organizational commitment emerges when employees recognize that there are limited comparable alternatives for them, in case they decide to leave the organization (Meyer et al., 1993).

Summarizing, “employees with strong affective commitment remain because they want to, those with strong continuance commitment because they need to, and those with strong normative commitment because they feel they ought to do so” (Allen and Meyer, 1990, p. 3).

### The Mediating Role of Organizational Commitment on the Relationship Between HPWS and Employee Performance

Previous literature has shown that HPWS can affect employee performance in different ways and, as a result, may influence organizational performance (Lepak et al., 2006; Takeuchi et al., 2009). A relatively small number of studies revealed a significant direct relationship between HPWS and employee performance (Kooij et al., 2013; Tabiu and Nura, 2013; Korff et al., 2017; Martinaityte et al., 2019). At the same time, previous literature has attempted to understand the mechanisms through which HPWS are related to employee performance, and several dominant perspectives have been used to explain the “black box” at the unit level of analysis in the strategic HRM literature (Messersmith et al., 2011; Jiang et al., 2013).

Regarding the effects of HPWS on organizational commitment, previous literature indicates that HRM practices can be crucial in this direction (Smeenk et al., 2006; Paré and Tremblay, 2007; Lee et al., 2018; Dorta-Afonso et al., 2021; Fragoso et al., 2021; Tej et al., 2021). According to Meyer and Allen (1991), the dominant predictor of affective commitment is, by far, work experiences. In particular, employees who satisfy their needs and whose experiences within the organization are consistent with their expectations, are more affectively attached to it than employees with less satisfying experiences (Meyer et al., 1993). The implementation of HPWS within an organization is considered to be a positive work experience for the employees, and a concrete signal of the company’s support and commitment toward them (Eisenberger et al., 1986), which is expected to positively influence affective commitment. Even though the majority of previous research evidence has identified positive links between HRM and affective commitment (Bos-Nehles and Meijerink, 2018; Fragoso et al., 2021), Meyer and Allen (1997) propose that HRM practices also affect normative and continuance commitment. The association between HPWS and the different components of organizational commitment can be supported by the social exchange theory (Blau, 1964). Social exchange emerges as a result of mutual reinforcement and trust (Blau, 1964; Aryee et al., 2002). According to this theory, employees will view HPWS as a valuable investment in them, to which they reciprocate through correspondingly positive attitudes and behaviors that benefit the organization (Hannah and Iverson, 2004; Dong and Zhong, 2021). As a result, it is expected that when employees receive positive treatment *via* effectively implemented HPWS, they will repay the organization through their increased levels of organizational commitment (Takeuchi et al., 2007). For example, when employees receive a training program or attractive benefits, they will perceive the organization as caring (developing strong affective commitment), they will feel a sense of indebtedness and obligation toward the organization (developing strong normative commitment) and they will believe that losing the provision of such practices would be costly for them (developing strong continuance commitment; Meyer and Smith, 2000). Based on the above, and building on SET, employees are expected to reciprocate to the provision of HPWS by their organization, with increased levels of affective, normative, and continuance commitment.

Regarding the relationship between organizational commitment and employee performance, previous literature demonstrated interesting findings, mainly for the affective component. Not only have several empirical works showed a positive association between the two variables (Meyer et al., 1989; Chang and Chen, 2011; De Cuyper and De Witte, 2011; Innocenti et al., 2013; Franco and Franco, 2017; Kloutsiniotis and Mihail, 2017), but meta-analyses (Meyer et al., 2002; Riketta, 2002) have also consistently reported positive and moderate correlations between the two variables. We argue that employees with high levels of affective commitment to the organization are likely to demonstrate high performance, due to their dedication and high level of involvement to it (Meyer et al., 1989; Chang and Chen, 2011). It is generally argued that affective commitment, and normative commitment to a lesser extent, should have the strongest correlations with organization and employee-related outcomes, including employee performance (Meyer et al., 2002; Vandenberghe et al., 2015). Some previous literature has identified significant links between normative commitment and employee performance (Cesario and Chambel, 2017). For the purpose of this study, we assume that employees with a strong sense of indebtedness and obligation will demonstrate high performance, because they perceive their organization as supportive and caring.

On the other hand, previous literature demonstrates that continuance commitment is unrelated, even related negatively to outcome variables, such as employee performance (Meyer et al., 1989, 2002). Continuance commitment has been also shown to positively relate with “dark” characteristics, such as narcissism (Kaufmann et al., 2021), that associate with counterproductive behaviors (O’Boyle et al., 2012). Highly continuance committed employees are expected to put the minimum possible effort, that will allow them to minimize their costs. In general, employees “who feel compelled to remain, to avoid financial or other costs, may do little more than the minimum required to retain their employment” (Meyer et al., 1989, p. 152). Based on the above, we argue that continuance commitment will not be related to employee performance.

Summarizing, a perceived implementation of effective HPWS will lead to greater affective attachment, stronger feelings of obligation toward the organization, and to sounder recognition of the costs of not being committed (Shore and Wayne, 1993). In turn, we expect that employees feel an obligation toward their employers and, as a result, are more likely to respond with positive work attitudes, developing an affective and normative bond with the organization itself. Subsequently, employees’ increased levels of affective and normative commitment are engender beneficial individual performance (Takeuchi et al., 2007; Cesario and Chambel, 2017). Conversely, we expect that employees with high levels of continuance commitment will not respond with high levels of employee performance, as their primary aim is to sustain their acquired benefits, instead of contributing to the organization itself or exerting extra-role performance behaviors. Following the line of reasoning above, hypotheses H1a, b, and c are formulated:

*H1a*: Affective commitment will positively mediate the relationship between HPWS and employee performance.

*H1b*: Normative commitment will positively mediate the relationship between HPWS and employee performance.

*H1c*: Continuance commitment will not mediate the relationship between HPWS and employee performance.

### The Moderating Role of Employee Tenure

Tenure is defined as the length of time that an employee has been in the employment of an organization (McEnrue, 1988). Findings demonstrate that employees with longer tenure usually accumulate more skills and experience (Yang et al., 2015), and show higher levels of in-role and extra-role performance (McEnrue, 1988; Ng and Feldman, 2010). Previous literature has examined tenure as a factor that can associate with employee outcomes (Oreilly and Caldwell, 1981; Yanadori and Kato, 2007; Ng and Feldman, 2010; Godfroid et al., 2022) or can act as a moderator in the link between organizational commitment and employee performance (Wright and Bonett, 2002), turnover (Taylor et al., 1996), leadership and job satisfaction (Baek et al., 2019). In addition, Hu et al. (2019), examined the role of organizational tenure in the relationship between perceived HPWS and affective organizational commitment, indicating that the effect of perceived HPWS on affective commitment is stronger among employees with longer tenure.

As mentioned above, in this study we adopt a social exchange perspective. According to SET (Blau, 1964), in the long term, separate exchanges create relations between parties (Cropanzano and Mitchell, 2005; Cropanzano et al., 2017). Repeated exchanges create reciprocation obligations, which also create new exchanges and recurring obligations. Eventually, repeated exchanges become the norm and form solid relations between exchanging parties. In the current study, we assume that there is a positive social exchange effect, which links provisions in HPWS with affective, normative, and continuance commitment. We expect that this positive exchange effect will be stronger, the deeper the relationship developed is. The length of the exchange relationship (i.e., employee tenure) is expected to affect the quality of the relationship established and subsequently the magnitude of the positive exchange effects.

More simply, we assume that employee tenure will act as a moderator on the relationship between HPWS and organizational commitment. We argue that an efficient implementation of practices within the organization will be more positively associated with affective, normative, and continuance commitment, the longer time employees have stayed with the organization, because the length of service stabilizes the relationships established during social exchanges. In particular, employees who are offered sound HPWS will reciprocate with higher affective commitment and will sincerely care more about the benefit of the employer, the more they have stayed in the organization. Also, they will reciprocate with higher normative commitment and will act on the benefit of the employer out of a sense of obligation, the more they have remained in the organization.

Finally, the longer employees have stayed with the company makes them realize the value of the relationship established and its benefits (for example good HPWS), meaning that they value more the opportunity cost of not being committed. Also, continuance commitment is a job attitude that is likely to be salient among aging employees (Luchak et al., 2008), who most probably are the ones with the longer tenure. For these, late-career employees, the costs of leaving the organization and the perceived lack of alternatives, are important motives that affect their decision to remain in the organization (Herrbach et al., 2009). We argue that employees with longer tenure will benefit from a fair implementation of HPWS, which will create “advantageous conditions they do not want to give up while experiencing less mobility in the market” (Herrbach et al., 2009, p. 898). Therefore, the more an employee has remained with an employer, the more he/she will appreciate provisions like HPWS, which he/she will be reluctant to jeopardize. Following the line of reasoning above, hypotheses H2a, b, and c are formulated:

*H2a*: Employee tenure will moderate the relationship between HPWS and affective commitment, so that the positive effect of HPWS on affective commitment will be stronger for long-term employees.

*H2b*: Employee tenure will moderate the relationship between HPWS and normative commitment, so that the positive effect of HPWS on normative commitment will be stronger for long-term employees.

*H2c*: Employee tenure will moderate the relationship between HPWS and continuance commitment, so that the positive effect of HPWS on continuance commitment will be stronger for long-term employees.

**Figure 1:**
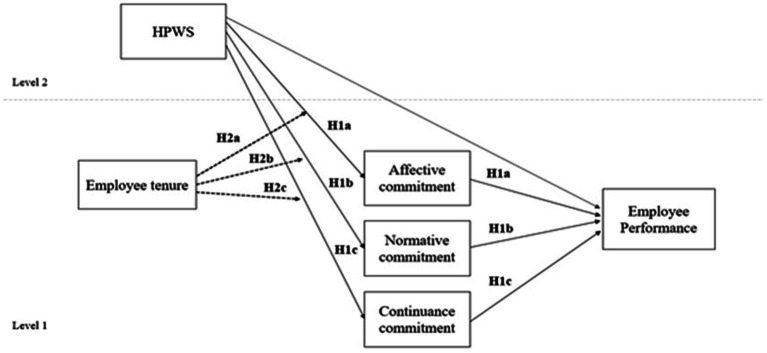
Conceptual framework.

Figure 1 below, represents the conceptual framework of the study.

## Materials and Methods

### Sample and Procedure

Our study was addressed to working groups in organizations operating in the Greek service sector, in industries, such as telecommunications, banking, education, and healthcare. For the realization of our multilevel study, we created two questionnaires, one addressed to supervisors, and one to subordinates. For the data collection, both the research team and selected students who were trained as researchers, followed clear instructions on how to contact the supervisors. We selected the organizations through random interviewer selection, based on restrictions that related to company type, as well as with supervisor and subordinate tenure (i.e., more than 6 months of working experience). Regarding the distribution of the questionnaires, we first contacted supervisors, who evaluated their subordinate’s performance. As a next step, once the supervisors agreed to answer the questionnaire, they then distributed the subordinate’s questionnaire, to the same subordinates that they evaluated while filling their questionnaire. In simple words, if the supervisor evaluated the performance for subordinates X, Y, Z, then subordinates X, Y, Z were distributed a second questionnaire, where they evaluated the implementation of HPWS in their organization, as well as their own levels of organizational commitment, and the number of working years for the current organization. Both the supervisors’ and the subordinates’ questionnaires were distributed and answered, either using a paper-and-pencil version or through e-mails. Anonymity was guaranteed during all steps of the procedure, as subordinates were assigned an identification number, and a black sticker was positioned over the specific field in the supervisors’ questionnaire. This way, the subordinates did not know the performance evaluation scores of their supervisors, neither could the latter identify how the former answered to their questionnaire.

The final sample consisted of 342 subordinates (57.9% female), with a mean age of 39.59 years (SD = 10.178) and an average tenure of 9.41 years (SD = 8.904) and 115 supervisors (65.2% male, mean age = 47.34 years) of 115 working groups, from 111 service organizations (in four out of the 111 organizations we collected responses from two working groups).

### Measures

#### High Performance Work Systems

For the measurement of HPWS, we adopted the Human Resources Management Policies and Practices Scale (Sun et al., 2007), which assesses eight HPWS with 27 items. HPWS were measured based on employees’ perceptions. Respondents evaluated the implementation of practices in their organization, on a five-point Likert scale, varying from 1 (I totally disagree) to 5 (I totally agree). “Internal Mobility” had a low reliability (*a* = 0.38), so we excluded it from our analysis and we used seven practices, namely “Selective staffing,” “Extensive Training,” “Employment security,” “Clear job description,” “Results oriented appraisals,” “Incentive reward,” and “Participation.” HPWS were measured as a single second-order factor, estimated as a mean score of the seven practices (*a* = 0.81).

For the measurement of HPWS at the group level, we aggregated subordinates’ perceptions, by analyzing the ICC_1_ and ICC_2_ to assess agreement among group members, as well as the inter-rater agreement (*r*_wg_). The values of intra-class correlation (ICC_1_ = 0.48 and ICC_2_ = 0.74) and inter-rater agreement (mean *r*_wg_ = 0.85) statistically justified the aggregation of HPWS at the group level (LeBreton and Senter, 2008).

#### Employee Performance

For the measurement of employee performance, we adopted the 20-item Role-based Performance Scale (Welbourne et al., 1998). The supervisors assessed their subordinates’ performance on 20 in-role and extra-role aspects of performance, on a scale ranging from 1 (needs much improvement) to 5 (Excellent). Sample items were “Quantity of work output,” “Obtaining personal career goals,” and “Coming up with new ideas.” The scale indicated a high and acceptable reliability (*a* = 0.91).

#### Organizational Commitment

For the measurement of organizational commitment, we used the Allen and Meyer commitment questionnaire (Allen and Meyer, 1990), which measures, with 24 questions, affective (eight items), normative (eight items), and continuance commitment (eight items). Employees evaluated their organizational commitment on a five-point Likert scale, varying from 1 (I totally disagree) to 5 (I totally agree). Sample items included “I enjoy discussing my organization with people outside it” (affective commitment), “I was taught to believe in the value of remaining loyal to one organization” (normative commitment), and “It would not be too costly for me to leave my organization now (rev)” (continuance commitment). The scale indicated acceptable reliability for the two aspects of organizational commitment (affective commitment: *a* = 0.845; continuance commitment: *a* = 0.727). However, the reliability score for normative commitment was 0.694. By removing the item “I think that people these days move from company to company too often,” reliability was increased to acceptable levels (*a* = 0.727). As a result, normative commitment was used as a 7-item construct for subsequent analyses.

#### Employee Tenure

Tenure was measured as a continuous variable in “number of years working for the current organization.”

#### Control Variables

Subordinates’ *gender* was coded as 1 for male and 2 for female. *Education* was a continuous variable, measured as the number of years of completed formal education. *Job type* was coded as 1 for white-collar employee and 2 for blue-collar employees. In addition, following previous research (Dries et al., 2009; Guan et al., 2014), *Job hierarchy* was estimated by dividing each subordinate’s absolute level by the total number of levels in the hierarchy of the organization. Finally, *age* was not included in our research model, due to its high correlation with employee tenure (*r* = 0.82), which could create multicollinearity issues.

## Results

Table 1 displays descriptive statistics, including the means, standard deviations, and correlations.

**Table 1:** Correlation matrix.

	M	SD	1	2	3	4	5	6	7	8	9	10
1. HPWS^a^	3.248	0.609	1									
2. Employee performance^b^	3.705	0.645	−0.012	1								
3. Affective commitment^c^	3.404	0.865	0.491^***^	0.158^***^	1							
4. Normative commitment^c^	3.324	0.767	0.358^***^	0.001	0.692^***^	1						
5. Continuance commitment^c^	3.391	0.748	0.072	0.091	0.278^***^	0.270^***^	1					
6. Employee tenure	9.409	8.904	−0.212^***^	0.129^**^	0.027	−0.027	0.241^***^	1				
7. Gender	1.580	0.494	−0.119^**^	0.127^**^	−0.051	−0.064	0.092^*^	0.087	1			
8. Education	14.060	2.808	−0.008	0.098^*^	0.047	−0.056	−0.119^**^	−0.107^**^	0.166^***^	1		
9. Job type	1.140	0.345	−0.013	−0.123^**^	−0.130^**^	−0.034	−0.136^**^	−0.140^***^	−0.244^***^	−0.296^***^	1	
10. Job hierarchy	0.508	0.266	0.096^*^	0.018	0.192^***^	0.134^**^	0.040	0.101^*^	−0.132^**^	−0.115^**^	0.027	1

a Aggregated subordinates’ perceptions.

b Supervisors’ ratings.

c Subordinates’ perceptions.

* *p* < 0.10;

** *p* < 0.05;

*** *p* < 0.01.

### Measurement Model

We conducted a series of confirmatory factor analyses (CFA), in order to examine the validity of our constructs. In particular, we examined five models, one for each of the key constructs of the research model (i.e., HPWS, affective commitment, continuance commitment, normative commitment, and employee performance). As presented in Table 2, our analysis showed acceptable fit indices for all our key variables.

**Table 2:** Confirmatory factor analysis.

Construct	*x* ^2^	df	*x*^2^/df	*p*	CFI	TLI	SRMR	RMSEA
HPWS	497.837	188	2.648	0.000	0.919	0.901	0.056	0.070
Employee performance	553.205	163	3.393	0.000	0.902	0.885	0.052	0.084
Affective commitment	43.080	20	2.154	0.000	0.977	0.966	0.029	0.058
Normative commitment	38.754	12	3.229	0.000	0.939	0.893	0.044	0.081
Continuance commitment	53.278	18	2.960	0.000	0.926	0.886	0.049	0.076
**Alternative models**								
5-factor model	3,992.618	1999	1.997	0.000	0.817	0.810	0.073	0.054
3-factor model	4,221.712	2006	2.104	0.000	0.797	0.789	0.072	0.057
1-factor model	9,375.808	2016	4.651	0.000	0.325	0.303	0.159	0.104

In addition, we ran a series of CFA for the hypothesized 5-factor model (HPWS, affective, normative, continuance commitment, employee performance) and for alternative models (see Table 2). When compared with alternative models, the 5-factor model indicated a better fit, than the 3-factor model (where affective, normative, and continuance commitment were grouped into one factor), and the 1-factor model (where affective, normative, and continuance commitment where grouped into one factor; Marsh and Hocevar, 1985).

### Data Analysis

In order to test our research model, we used multilevel structural equation modeling (MSEM) with the STATA 14 software. In our research model, we include a random intercept in each equation at the employee (individual) level. Before running the analysis, all variables were grand mean centered. Grand mean centering is considered to be an appropriate method for mediational models, where “group-level variables influence individual behaviors and attitudes only indirectly through other mediating mechanisms” (Hofmann and Gavin, 1998, p. 635).

We also checked for multicollinearity and heteroscedasticity by estimating the variance inflation factor (VIF) and by performing the Breusch–Pagan test. All predictors had values lower than 2.14, which are below the maximum threshold of 5 (Hair et al., 1995), indicating that multicollinearity was not an issue for our research model. However, given the wide evidence indicating a high correlation between normative and affective commitment (Meyer et al., 2002), and based on the high correlation between the two variables in the current data set (*r* = 0.692), we added a covariance term between affective and normative commitment in our model, to better grasp the total relationships developed. Regarding the heteroscedasticity test, our results showed a chi-square value of 0.39 and a value of *p* of 0.534 (*p* > 0.005). As a result, the null hypothesis which stated that there is a constant variance among the residuals was not rejected, meaning that heteroscedasticity was not present in our data.

### Hypotheses’ Testing

Results of the MSEM analysis are presented in Table 3 below.

**Table 3:** Multilevel structural equation modeling analysis.

Independent variables	Affective commitment	Normative commitment	Continuance commitment	Employee performance	
				Model 1	Model 2
Gender	−0.021	−0.029	0.112	0.059	0.061
Education	0.001	−0.016	−0.039^***^	0.007	0.005
Job type	−0.237^**^	−0.011	−0.282^**^	−0.196^*^	−0.157
Job hierarchy	0.401^***^	0.247^*^	0.008	0.117	0.035
HPWS	0.617^***^	0.423^***^	0.152^**^	−0.003	−0.047
Affective commitment					0.193^***^
Normative commitment					−0.124
Continuance commitment					−0.036
Employee tenure	0.012^***^	0.004	0.019^***^		0.005
HPWS*Tenure	0.021^***^	0.014^*^	0.002		
var. (M1[Company_ID])	1.256	0.650	1.000	0.197	7.316
var. (e.Performance)	0.201	0.201	0.201	0.209	0.201
var. (e.Affective)	0.432	0.432	0.432		0.432
var. (Normative)	0.496	0.496	0.496		0.496
var. (e.Continuance)	0.496	0.496	0.496		0.496
cov (Affective, Normative)	0.286^***^	0.286^***^	0.286^***^		0.286^***^
Log-likelihood	−1,276.366	−1,276.366	−1,276.366	−294.278	−1,276.366

*n* = 342 individuals nested in 115 groups.

* *p* < 0.10;

** *p* < 0.05;

*** *p* < 0.01.

Regarding the effects of the control variables, job type was negatively associated with affective (*b* = −0.237, *p* < 0.05) and continuance commitment (*b* = −0.282, *p* < 0.05), showing that white-collar employees indicate higher levels of affective and continuance commitment than blue-collar employees. Job type was also found to negatively and significantly relate with employee performance (*b* = −0.196, *p* < 0.10), indicating that white-collar employees demonstrate higher levels of performance than blue-collar employees. In addition, job hierarchy was positively and significantly related to affective (*b* = 0.401, *p* < 0.01) and normative commitment (*b* = 0.247, *p* < 0.10), showing that employees at higher hierarchical levels are more emotionally attached with the organization and have a higher sense of obligation toward the organization. Moreover, education was negatively associated with continuance commitment (*b* = −0.039, *p* < 0.001), showing the employees with more years of formal education, are less likely to remain in the organization because of the fear of losing the benefits associated with their employment. Employee tenure was found to be positively related to affective commitment (*b* = 0.012, *p* < 0.001), and continuance commitment (*b* = 0.019, *p* < 0.001). The above findings demonstrate that long-term employees are more committed to the organization in terms of those two aspects.

Regarding the mediation analysis, there was no significant direct effect of HPWS on employee performance (*b* = −0.003, *p* > 0.10) when organizational commitment was not part of the analysis. Also, HPWS did not significantly and directly affect employee performance (*b* = −0.047, *p* > 0.10) when the commitment mediators were included in the analysis. Our results showed that the effect of HPWS on affective commitment was positive and significant (*b* = 0.617, *p* < 0.001) and that affective commitment was positively associated with employee performance (*b* = 0.193, *p* < 0.01). Table 4 also shows that there is a significant indirect effect of HPWS on employee performance, through affective commitment (*b* = 0.119, *p* < 0.05). Building on previous scholars, we argue that “attention in mediation analysis should be shifted toward assessing the magnitude and significance of indirect effects” (Rucker et al., 2011, p. 359). As a result, the insignificant total effect (*b* = 0.072, *p* > 0.10) is not a prohibitive condition for justifying the mediation hypotheses. Therefore, affective commitment fully mediates the relationship between HPWS and employee performance, herewith supporting Hypothesis H1a.

**Table 4:** Indirect and total effects.

Effect	Coef.	Std. err.	*z*	*p*	95% conf. interval	
Indirect through affective commitment	0.119	0.053	2.25	0.024	0.015	0.222
Total	0.072	0.088	0.82	0.414	−0.100	0.244

Regarding the mediating effect of normative commitment, HPWS were positively related to normative commitment (*b* = 0.423, *p* < 0.001). However, normative commitment was not significantly associated with employee performance (*b* = −0.124, *p* > 0.10). Therefore, the mediating Hypothesis H1b is rejected. Additionally, even if HPWS were positively associated with continuance commitment (*b* = 0.152, *p* < 0.05), continuance commitment was not significantly associated with employee performance (*b* = −0.036, *p* > 0.10). Therefore, Hypothesis H1c which hypothesized that there is not any mediating effect of continuance commitment on the association between HPWS and employee performance was supported.

**Figure 2:**
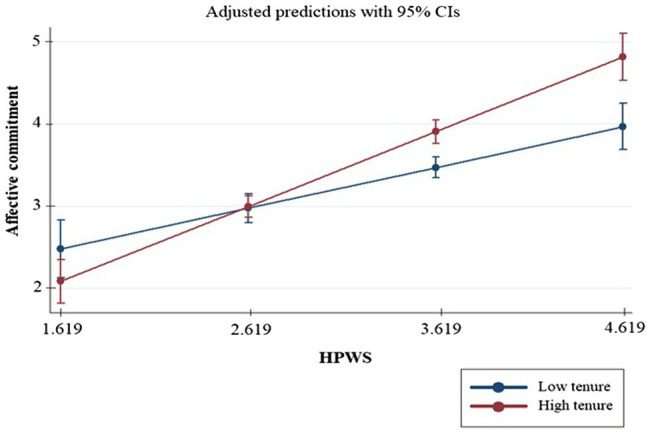
Interaction plot—affective commitment.

**Figure 3:**
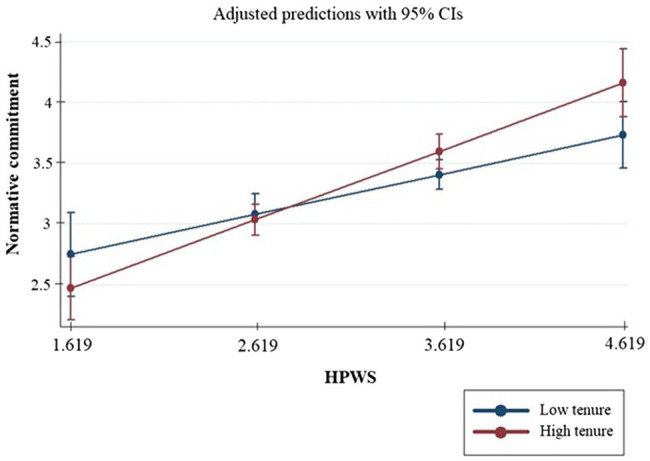
Interaction plot—normative commitment.

Regarding the moderating hypothesis, employee tenure had a positive effect on the relationship between HPWS and affective commitment (*b* = 0.021, *p* < 0.001), herewith supporting Hypothesis H2a. This finding indicates that the implementation of HPWS leads to increased levels of affective commitment for long-term employees, more than it does for employees with lower tenure. Similarly, employee tenure positively moderated the link between HPWS and normative commitment, supporting Hypothesis H2b (*b* = 0.014, *p* < 0.10) and showing that the effect of HPWS on normative commitment is stronger for employees with longer tenure. On the other hand, employee tenure did not moderate the association between HPWS and continuance commitment (*b* = 0.002, *p* > 0.10), therefore rejecting Hypothesis H2c. The effect of HPWS on continuance commitment does not change with tenure. To better understand the character of this interaction, we plotted the regression lines for the relationship between affective and normative commitment and the aggregated perceptions of HPWS at 1 SD below and 1 SD above the mean employee tenure. Figure 2 shows that the association between HPWS and affective commitment is stronger for long-term employees (*b* = 0.802, SE = 0.088, *p* = 0.000) than for the short-term ones (*b* = 0.434, SE = 0.089, *p* = 0.000). Moreover, Figure 3 also shows that the relationship between HPWS and normative commitment is stronger for long-term employees (*b* = 0.547, SE = 0.095, *p* = 0.000) than for short-term ones (*b* = 0.300, SE = 0.095, *p* = 0.002).

## Discussion

Our study contributes to the SHRM literature in different ways. First, we adopted the universalistic or “best practices” view (Delery and Doty, 1996), according to which specific sets of HRM practices and mechanisms are beneficial to all organizational settings. According to this approach, by adopting a universal set of good HRM practices (Guest, 1997), organizations can benefit from increased affective commitment and performance at the individual level. The findings suggest important cross-level mechanism effects between organizational-level perceptions of HPWS, organizational commitment, and employee performance. It should be noted though, that according to our findings, HPWS enhance employee performance only through commitment, not directly. This puts much emphasis on the feelings that organizational practices create and supports our decision to adopt the SET framework to explain the relationship between HPWS and performance. Our study adds to the discussion on the “black box” in SHRM (Jiang et al., 2013) by suggesting that HPWS can lead to increased individual performance through the intervention of mediating factors. Our study indicates that there is not a direct link between organizational HPWS and employee performance and that this relationship is indirect, through the mediating effect of affective commitment.

Our findings also contribute in theory by building on a social exchange perspective (Blau, 1964). Our study showed that HPWS have significant and direct effects on all types of organizational commitment. Under the lens of SET and the norm of reciprocity (Gouldner, 1960), this finding shows that when employees have positive perceptions on the implementation of HPWS in their organization, they reciprocate by demonstrating increased levels of affective, normative, and continuance commitment. For example, when employees receive proper training programs, attractive compensation packages, and fair appraisal and staffing, they are committed to the organization, either because of an emotional attachment (affective), or a feeling of obligation (normative), or a consideration of the risk to compromise vested interests in case of no commitment (continuance). This finding supports previous literature that shows positive effects of HRM practices or HPWS and different types of organizational commitment (Meyer and Allen, 1997; Smeenk et al., 2006).

However, our study only partly confirms that positive employees’ perceptions of a supporting work environment, oblige them to respond with positive and beneficial for the organization behaviors (Sun et al., 2007). The results show that HPWS can yield positive employee performance by fostering only employees’ affective commitment. The confirmation of Hypothesis H1a supports previous literature findings, showing that the implementation of HPWS can increase an employee’s affective commitment and subsequently his/her performance (Chang and Chen, 2011; Dorta-Afonso et al., 2021; Fragoso et al., 2021). On the other hand, normative and continuance commitment were not shown to mediate the HPWS—employee performance relationship, hereby rejecting Hypothesis H1b and supporting Hypothesis H1c. These findings align with previous literature which shows that continuance commitment does not relate with employee performance and that normative commitment has weak links with job outcomes (Meyer et al., 1989, 2002; Fragoso et al., 2021). Also, this supports recent allegations on the criticality of emotions and affect for positive employee attitudes and outcomes (Mostafa, 2017). In the SET literature, there is a lot of emphasis on the reciprocation effect, that is, when one receives positive treatment, she wants to repay the favor with positive acts. Our study shows that employees do reciprocate with positive attitudes (affective, normative, and continuance commitment), but the positive attitude is translated into positive behavior (employee performance), only when the positive attitude supported is affective commitment. In other words, HPWS enhance performance only when they make employees care for the organization’s benefit (a “pull” factor), not out of a feeling of obligation (normative commitment), nor out of fear of losing benefits (continuance commitment; both “push” factors).

In addition, our study investigated the moderating role of tenure in the relationship between HPWS and organizational commitment, suggesting positive moderating effects for all three types of commitment. The confirmation of Hypothesis H2a, predicting that there is a positive moderating effect of tenure in the relationship between HPWS and affective commitment, supports previous literature findings (Hu et al., 2019). Therefore, the longer time employees have spent with an organization, the more, positive organizational provisions (HPWS) foster a feeling of belonging and caring (i.e., affective commitment). Similarly, the confirmation of Hypothesis H2b shows that the longer employees have stayed in the organization, the more they reciprocate to HPWS with normative commitment, that is, they feel the obligation to be committed. These two positive moderating effects could be linked with a signaling confirmation effect: HPWS lead to affective and normative commitment, the longer employees have experienced them and developed trust in the organization and its practices. Also, as SET defines it (Cropanzano et al., 2017), social exchanges lead to the creation of a relation between the two parties, and relations take long to develop.

On the other hand, tenure was not found to moderate the link between HPWS and continuance commitment, herewith rejecting Hypothesis H2c. Specifically, no matter how long employees have stayed with the organization, they will reciprocate to HPWS with the same level of continuance commitment, that is, they will be committed to the same extent, out of fear of losing the benefit of HPWS. This finding contradicts previous literature which shows that late-career employees are more cautious of unemployment and career risks than their more junior counterparts, because they generally benefit more from advantageous conditions (for example higher salaries or higher positions) that they do not want to compromise (Herrbach et al., 2009) and they feel that their employability and alternative options are more finite.

Finally, our study also confirmed that tenure associates with organizational commitment, supporting the argument that commitment varies throughout employees’ careers (Conway, 2004). Specifically, employee tenure was directly and positively associated with affective and continuance commitment, aligning with previous literature findings (Cohen, 1999). Long-term employees are more emotionally attached to the organization than recent hires. Also, employees who have been working for a long time in the same organization perceive the costs of leaving as higher than recent hires do, and have higher continuance commitment. This is most probably related to the advantages that high tenure entails for employees, for example, feeling at ease, knowing the people and the work, learning effects and high productivity, feeling of security, possible higher wages, and opportunities for development, which they are reluctant to put at risk.

## Practical, Economic, and Societal Implications

Employers see HRM practices as investments, on which they seek to maximize return. HPWS constitute an extensive investment in human resources, therefore considerable positive business outcomes are expected in return. An implicit assumption during the HPWS design is that HPWS will repay their cost through increased employee performance, at the individual, department, and business level. This study’s findings provide evidence on the link of HPWS with individual employee performance, therefore its practical implications mostly focus on the returns of the HPWS implementation. In addition, managers who search for ways to increase the individual performance of employees, and especially the underperforming ones, should focus on the implementation of fair HPWS (e.g., staffing, training, and participation). The implementation of such practices may increase employees’ emotional attachment to the organization, which will eventually lead to increased performance outcomes.

Based on our findings, HPWS implementation pays more in the case of long-tenured employees. Allowing or encouraging employees to stay longer should therefore be a quest for all employers, if not already. Certainly, HPWS are the main tool that increases commitment (Qiao et al., 2009); therefore, a spiral effect/beneficial cycle seems to exist in this respect (HPWS lead through tenure to high commitment and then commitment leads to high tenure, which further fosters commitment). However, there are other things that can improve the retention of employees, prolong length of service and therefore increase the performance outcomes of HPWS, such as enhancement of organizational culture and identification, good communication channels, and sound organizational structures. Organizations are advised to couple HPWS with several other practices that reduce employee turnover and prolong employee tenure. In addition, HRM departments are advised to invest in the implementation of organizational practices that are targeted toward long-tenured employees (e.g., flexible arrangements, part-time work, leadership training programs, training on IT skills, and mentoring programs). Implementing practices that fit with the needs and characteristics of long-tenured employees may lead to reciprocal commitment behaviors.

What is more, attention should be paid on explicitly communicating the application of HPWS to shorter tenured employees, especially newcomers. As the reciprocation effect to HPWS seems to be enhanced the longer employees have remained in the organization and familiarized with HPWS practice, it could be helpful to further familiarize new employees with organizational practices, so that they value and start reciprocating to them as early as possible.

This study has emphasized once more, the criticality of affective commitment in comparison to other types of commitment for the maximization of performance outcomes. Many employers keep on using coercion, intimidation, and stress (all of them push factors), as ways to enhance employee performance, even if these are prone to foster primarily continuance commitment. Our study affirms that only through affective commitment do the costly HPWS pay off. Affective commitment is fostered with positive feelings, such as safety, respect, care, concern, support in difficulty, acceptance of mistakes, and inclusion, in other words, treatment that allows employees to “feel at home” and develop affect and genuine concern for their organization (pull factors).

Our study did not show mediating effects of normative and continuance commitment in the HPWS-performance link. In other words, committed (in terms of normative and continuance dimensions) employees do not reciprocate with positive performance behaviors. Therefore, organizations should find ways to increase the performance of those employees. Creating a culture of trust and teamwork, applying job rotation practices, and encouraging individual creativity and innovation could be some useful recommendations toward this direction.

Our study findings also provide economic and societal implications. First, this study demonstrates the importance of implementing HPWS in the context of service firms in Greece. Given the huge impact of the service sector on the national economy (i.e., service sector accounts for almost 70% of the national GDP in the 2010–2020 decade—Statista, 2022), our results indicate that the application of HPWS in service organizations associates with increased performance outcomes, which at a macro-level, can contribute to the national economic development. Also, the increased formation of human capital and/or organizational commitment at an aggregate level can improve employee performance and therefore, can raise the *per capita* income. Our results also show that HPWS facilitate generalized norms of reciprocity, through increased levels of employees’ organizational commitment. Encouraging sustainable professional relationships between organizations and employees, where the rewards are higher than the costs of the relationship, can benefit the organization and the society as a whole. Finally, long-term employees that remain in the same organization for many years, continue to learn, to be motivated, and to be productive. Embracing a culture of lifelong learning and development can be an important step toward a societal contribution.

## Limitations and Future Research

The current research has several limitations related to the measurement of variables and the context of the study, which open new directions for future research.

First, as part of our research methodology, we collected cross-sectional data from service organizations operating in Greece. The nature of our study prohibits inferring cause–effect relationships. Future research could adopt a longitudinal design to uncover how the links between HPWS, organizational commitment, and employee performance develop and unfold over time.

Second, this study conceptualized and tested HPWS as a system (Sun et al., 2007), through the lens of a universalistic approach in HRM, to illustrate the mediating roles of affective, normative, and continuance commitment. However, it is possible that some subcomponents of HPWS have differential impacts on specific types of commitment. For example, employee participation in decisions may increase employees’ affective commitment more than selective staffing. Similarly, incentive rewards may increase employees’ continuance or normative commitment more than results oriented appraisals. Future research could investigate those mediating mechanisms by focusing on individual HRM practices, instead of synergies of practices.

Third, for the purpose of this study, we performed a multilevel study by surveying 342 employees from 115 teams. Reaching this number of subjects and getting access to organizational groups, required substantial effort and resources. However, we acknowledge that this sample may not be the best possible in terms of the quantity of observations. As a result, our findings are not generalizable to the study population.

In addition, this study adds to the debate on how HPWS can lead to performance, by demonstrating an indirect effect through affective commitment and that HPWS can foster normative and continuance commitment without leading to performance. Future research could explore how the performance of employees with low affective commitment and high normative and continuance commitment can be enhanced.

Finally, our study contributes to the underexplored field of tenure and employee outcomes, by providing both moderating and direct effects on different types of organizational commitment. A future avenue for research is to explore how long-term employees across different employment stages can remain productive and prove that they are valuable assets for their organizations.

## Data Availability Statement

The datasets presented in this study can be found in online repositories. The names of the repository/repositories and accession number(s) can be found at: https://zenodo.org/record/4572943#.YaTRkvnMI2w10.5281/zenodo.4572943.

## Ethics Statement

Ethical review and approval was not required for the study on human participants in accordance with the local legislation and institutional requirements. Written informed consent for participation was not required for this study in accordance with the national legislation and the institutional requirements.

## Author Contributions

NP and EG conceived of the presented idea, developed the conceptual framework, were responsible for designing the study and collecting the data, and contributed in the discussion, implications, and limitations sections. NP analyzed the data in terms of descriptive statistics and multi-level analyses and carried out the literature review and hypotheses’ development for the mediating hypothesis. EG carried out the literature review and hypotheses’ development for the moderating hypothesis. All authors discussed the results and contributed to the final manuscript.

## Funding

The study was financed by a scholarship for doctoral studies funded by the General Secretariat for Research and Technology (GSRT) and the Hellenic Foundation for Research and Innovation (HFRI).

## Conflict of Interest

The authors declare that the research was conducted in the absence of any commercial or financial relationships that could be construed as a potential conflict of interest.

## Publisher’s Note

All claims expressed in this article are solely those of the authors and do not necessarily represent those of their affiliated organizations, or those of the publisher, the editors and the reviewers. Any product that may be evaluated in this article, or claim that may be made by its manufacturer, is not guaranteed or endorsed by the publisher.
